# Loss of Nogo receptor homolog NgR2 alters spine morphology of CA1 neurons and emotionality in adult mice

**DOI:** 10.3389/fnbeh.2014.00175

**Published:** 2014-05-15

**Authors:** Sarah C. Borrie, Simone B. Sartori, Julian Lehmann, Anupam Sah, Nicolas Singewald, Christine E. Bandtlow

**Affiliations:** ^1^Division of Neurobiochemistry, Biocenter, Innsbruck Medical UniversityInnsbruck, Austria; ^2^Department of Pharmacology and Toxicology, Institute of Pharmacy and Centre for Molecular Biosciences Innsbruck, University of InnsbruckInnsbruck, Austria

**Keywords:** Nogo receptor, NgR2, dendritic spine, hippocampus, anxiety, fear conditioning

## Abstract

Molecular mechanisms which stabilize dendrites and dendritic spines are essential for regulation of neuronal plasticity in development and adulthood. The class of Nogo receptor proteins, which are critical for restricting neurite outgrowth inhibition signaling, have been shown to have roles in developmental, experience and activity induced plasticity. Here we investigated the role of the Nogo receptor homolog NgR2 in structural plasticity in a transgenic null mutant for NgR2. Using Golgi-Cox staining to analyze morphology, we show that loss of NgR2 alters spine morphology in adult CA1 pyramidal neurons of the hippocampus, significantly increasing mushroom-type spines, without altering dendritic tree complexity. Furthermore, this shift is specific to apical dendrites in distal CA1 stratum radiatum (SR). Behavioral alterations in NgR2^−/−^ mice were investigated using a battery of standardized tests and showed that whilst there were no alterations in learning and memory in NgR2^−/−^ mice compared to littermate controls, NgR2^−/−^ displayed reduced fear expression in the contextual conditioned fear test, and exhibited reduced anxiety- and depression-related behaviors. This suggests that the loss of NgR2 results in a specific phenotype of reduced emotionality. We conclude that NgR2 has role in maintenance of mature spines and may also regulate fear and anxiety-like behaviors.

## Introduction

The ability of the brain to remodel, via structural and synaptic plasticity of dendrites and spines, is critical for development of functional circuits, as well as adaptive alterations in adulthood. Experience-dependent remodeling of synaptic connections occurs after the peak of synaptogenesis, during well-defined critical periods of postnatal development (Hensch, 2004). After the end of the critical period, structural plasticity in the adult brain is greatly decreased in response to experience-dependent changes (Lendvai et al., 2000; Majewska and Sur, 2003). Dendrites and spines in the adult cortex remain stable for long periods of time (Trachtenberg et al., 2002; Holtmaat et al., 2005), but remodeling of spines is still able to occur, as seen in the ability of small fraction of spines to turn over in long term *in vivo* imaging studies (Holtmaat et al., 2005, 2006). Facilitation of new learning has been linked to increased spine formation in behavioral studies. Spatial learning and associative learning have been reported to result increased hippocampal dendritic spines (Moser et al., 1994; Leuner et al., 2003). Another example of experience-dependent remodeling that can alter hippocampal spine and synapse formation is stress exposure (Shors et al., 2001; Pawlak et al., 2005; Donohue et al., 2006). Moreover, increasing evidence links alterations in synaptic plasticity to pathological conditions. Dendritic spine and dendritic branch loss are seen in psychiatric disease in the human brain, and are linked to impairments in cognition and emotional behaviors (Lin and Koleske, 2010).

The Nogo receptor (NgR) family and their ligands have been implicated in regulating forms of experience- dependent plasticity in the adult brain (Akbik et al., 2012). NgRs are a family of neuronally expressed glycophosphatidylinositol (GPI)-anchored leucine-rich repeat proteins, initially identified through their binding to a range of myelin-associated ligands inhibitory to neurite outgrowth in the CNS (Fournier et al., 2001; Domeniconi et al., 2002; Liu et al., 2002; Venkatesh et al., 2005). Nogo receptor 1 (NgR1) has been shown to regulate structural plasticity in the adult system. Knockout of NgR1 extends the critical period for experience-dependent plasticity in the ocular dominance paradigm in visual cortex (McGee et al., 2005), as well as extending critical period plasticity for acoustic preference (Yang et al., 2012). In addition, various forms of neuronal activity induction have been demonstrated to rapidly downregulate NgR1 expression in mice, as seen with wheel running (Josephson et al., 2003), kainic acid (Josephson et al., 2003; Mingorance et al., 2004; Wills et al., 2012; Karlsson et al., 2013), electroconvulsive seizures (Nordgren et al., 2013) and amphetamine (Guo et al., 2013). Regulation of the Nogo receptor homologs NgR2 and NgR3 by neuronal activity have also been reported (Wills et al., 2012; Karlsson et al., 2013). In support of this role in activity-dependent plasticity, NgR1 has been demonstrated to regulate synaptic plasticity. Ablation or blocking of NgR1 enhances hippocampal long-term potentiation (LTP) in slice preparations (Lee et al., 2008; Delekate et al., 2011), and appears to be mediated by the binding of NgR1 to selected ligands such as Nogo-A (Raiker et al., 2010), and fibroblast growth factor-2 (FGF2) (Lee et al., 2008). The extension of the critical period plasticity into adulthood is also reflected in an increase in spine turnover in the cortex of adult NgR1^−/−^ mice (Akbik et al., 2013), and a shift toward immature spine subtypes in the hippocampus of NgR1^−/−^ mice (Lee et al., 2008; Zagrebelsky et al., 2010). At a behavioral level, modulation of NgR1 has resulted in mild memory phenotypes. Forebrain NgR1 overexpression impairs long-term memory, without affecting short-term memory (Karlen et al., 2009), whilst constitutive knockout of NgR1 impairs working memory without affecting spatial memory (Budel et al., 2008). Additionally, NgR1^−/−^ mice also exhibit improved extinction learning in cued conditioned fear (Akbik et al., 2013). This suggests that NgR1-mediated structural plasticity may underlie some forms of behavioral plasticity, but highlight the potential functional redundancy of these receptors.

Thus far it is not known if the Nogo receptor homolog NgR2 can also function to restrict adult plasticity. NgR2 mRNA is highly expressed in hippocampal principal neurons of CA1, CA2, CA3 and dentate gyrus (Laurén et al., 2003; Barrette et al., 2007; Funahashi et al., 2008; Karlsson et al., 2013). NgR2 expression is also detected in neurons of all layers of the cortex (Laurén et al., 2003; Barrette et al., 2007; Funahashi et al., 2008; Karlsson et al., 2013) and of the amygdaloid complex (Barrette et al., 2007; Karlsson et al., 2013). It has been demonstrated that NgR1–3 can individually restrict excitatory synapse formation *in vitro*, and that loss of all 3 receptors increases dendritic length and excitatory synapse formation at postnatal day (P)18 (Wills et al., 2012). Here we investigated the individual function of NgR2 using an NgR2 mutant mouse line. We examined hippocampal morphology in the adult and report that ablation of NgR2 leads to regional specific increases in mushroom-type spines whilst leaving dendritic morphology normal. Additionally, NgR2^−/−^ mice exhibit alterations in fear and anxiety-like behaviors. We propose that NgR2 has a role in maintaining spine morphology in the adult system, which may be coincident with behavioral plasticity.

## Materials and methods

### Ethics statement

All animal experiments performed were approved by the local Ethical Committee on Animal Care and Use (Bundesministerium für Wissenschaft und Kultur, Austria; approval ID: BMWF-66.008/0016-II/3b/2011; BMWF-66.011/0166-II/3b/2012), in compliance with international laws and regulations for animal experimentation to minimize suffering and reduce the number of animals required.

### Animals

NgR2 null mutant mice were generated as described previously (Wörter et al., 2009), and backcrossed onto a C57BL/6J background for at least seven generations. All experiments were carried out on adult male littermate mice generated from NgR2 heterozygous breeding. Animals were group housed with littermates in cages under standard laboratory conditions (12 h light/dark cycle with lights on at 7 am, 22 ± 1°C, 60% humidity) with access to food and water *ad libitum*. All animals were genotyped by PCR of tail tissue.

### Morphological analysis

#### Nissl staining and immunohistochemistry

Naïve adult NgR2^+/+^ and NgR2^−/−^ littermate mice (*n* = 4 per genotype) were deeply anesthetized with an overdose of sodium pentobarbital and transcardially perfused with 0.9% saline followed by 4% paraformaldehyde in phosphate-buffered saline. Free-floating 40 μm sections were prepared on a cryotome (Leica) and either fixed on slides and processed for cresyl violet staining, or processed for immunohistochemistry with primary antibody for NeuN (anti-mouse NeuN, Chemicon), followed by biotinylated secondary antibody, amplification with Vectastain ABC reagent (Vector Laboratories) and development with 3,3'-diaminobenzidine chromagen. For neuron number analysis, 1 in 6 sections between Bregma −1.34 and −2.46 mm were analyzed for NeuN. Images from the dorsal hippocampus were obtained using an Olympus BX51 microscope and a 20× objective. A square region of 40,000 μm^2^ was placed over the CA1 pyramidal layer on the image and number of NeuN positive cells in the pyramidal layer counted, according to methods previously reported (Luikart et al., 2005). Data from 3 sections per brain were averaged together.

#### Golgi-cox staining and analysis

Naïve adult NgR2^+/+^ and NgR2^−/−^ littermate controls (*n* = 7 per genotype) were used for morphological analysis of CA1 pyramidal cells, using the Golgi-Cox method as previously described (Champagne et al., 2008). Briefly, freshly dissected brains were immersed in Golgi-Cox fixative solution for 28 days in the dark. Samples were then dehydrated and embedded in celloidin. Coronal sections 120 μm thick were cut on a sliding microtome. For staining, all sections were incubated in 16% ammonia solution to develop the impregnation, fixed in 1% sodium thiosulfate, dehydrated in ethanol, cleared in butanol and histoclear and mounted on slides.

Measurements of dendritic morphology were performed on 8–11 pyramidal cells per animal randomly chosen from the CA1 region of the dorsal hippocampus, defined as Bregma −0.94 to −2.80 mm (Franklin and Paxinos, 2008), by an observer blinded to genotype. Neurons were selected for tracing if they meet the following previously published criteria (Champagne et al., 2008): clearly filled, dendritic branches without breaks, and without precipitate. Neurons were traced using the Neurolucida program (MBF Biosciences, v10) attached to an Olympus BX51 microscope, using a 40× objective. Sholl and branching analysis of traced neurons was performed with Neurolucida Explorer program (MBF Biosciences).

For dendritic spine analysis, tertiary dendrites from the same CA1 pyramidal neurons were imaged with a 100× oil objective. Segments approximately 20 μm long were analyzed from each branch, by an observer blinded to genotype. Two to six tertiary segments per neuron were analyzed. Segments were analyzed from two regions of CA1 stratum radiatum (SR): 30–120 μm from the soma, and 120–300 μm from the soma. A spine had to be continuous to the dendritic shaft in order to be counted. The Neurolucida program (MBF Biosciences, v10) was used to assign spine categories to each spine, using previously described criteria for thin, stubby or mushroom spines. Density was determined by dividing number of spines on a segment by the length of that segment. Mean spine densities were obtained by averaging values per neuron, a widely accepted method of analysis in Golgi studies of dendritic morphology (Vyas et al., 2002; Chakravarthy et al., 2006; Pillai et al., 2012).

### Behavioral analysis

Adult NgR2^+/+^, NgR2^+/−^, and NgR2^−/−^ littermate mice were subjected to a battery of behavioral tests. For each test *n* = 8–16 mice per genotype were tested.

#### Morris water maze

In a circular pool (1.2 m in diameter) filled with opaque water (22°C) and illuminated at 70 lux, an escape platform (10 cm diameter) was hidden just below the water surface. Starting from variable positions, mice were placed into the water from which they could escape by climbing onto the platform. In case the animal was not able to locate the platform within 60 s, it was gently guided to it by the experimenter. The animals were allowed to stay on the platform for 30 s. On each of two consecutive training days, animals performed four trials separated by 60 min prior to probe trials 1 and 2 and probe trial reversal learning. The platform always remained in the same quadrant for each training session, but in the opposite quadrant for reversal learning while it was removed for the probe trials. In probe trials animals were allowed to swim in the pool for 60 s and the time the mice spent in the target quadrant was recorded.

#### Contextual fear conditioning

Contextual fear conditioning was performed as previously described (Sartori et al., 2011), in a fully automated fear-conditioning system (TSE, Technical & Scientific Equipment GmbH, Bad Homburg, Germany) consisting of a Perspex arena (23 × 23 × 35 cm) and a metal grid floor. For conditioning, mice were placed into the brightly illuminated (300 lux) context and 3 unsignaled mild foot shocks (0.6 mA, 2 s; US) were delivered. Two-minutes stimulus-free periods preceded, separated and followed the US presentations. Twenty-four hours after fear conditioning, mice were returned to the same context for 3 min to assess fear expression. The arena was thoroughly cleaned with tap water between each animal. All sessions were recorded via individual video cameras mounted above each context. Freezing behavior, defined as the absence of all non-respiratory movements (Blanchard and Blanchard, 1969; Fanselow, 1980), was taken as the measure of fear and was scored by an experienced investigator blinded to mouse lines and treatments. Percentages of freezing time for each mouse were calculated during each 2-min period post US presentation in the conditioning and during the 3-min context exposure in the fear expression tests. Six out of 42 mice in different experimental groups were insufficiently conditioned as they showed less than 10% freezing behavior after the final US presentation, and were therefore removed from the data set.

#### Flinch/jump test

The flinch/jump test was performed according to a previous protocol (Sartori et al., 2011). Animals were individually placed in the conditioning chambers (see above). After 2 min of habituation to the chamber, animals were subjected to 1 s shocks of gradually increasing amperage (0.1 mA every 30 s) starting at 0.1 mA. Mice were scored for their first visible response to the shock (flinch), their first pronounced motor response (run or jump), and for their first vocalized distress.

#### Open field

The open field consisted of a plastic box (41 × 41 × 41 cm) equipped with an automated activity monitoring system (TruScan, Coulbourn Instruments, USA). The area of the open field, illuminated with 150 lux, was divided into a 28 × 28 cm central zone and a surrounding border zone. Mice were placed individually into the periphery of the open field and allowed to explore it for 10 min. The following anxiety-related parameters were recorded: entries into the central zone, time spent in it and the overall distance traveled (Sartori et al., 2011).

#### Tail suspension test

The tail suspension test was performed as previously described (Schmuckermair et al., 2013). Mice were fastened with medical adhesive tape by the tip of the tail to a flat metallic surface and suspended for 6 min approximately 30 cm above the floor surface. Illumination was set at 100 lux. The activity of the mice was videotaped, and immobility, defined as when the animal hung passively without limb movement, was scored over the entire 6 min test session by a trained observer blinded to genotype. Data from the last 4 min of the test was analyzed.

#### Rotarod

The apparatus consisted of a rotating rod (diameter 3 cm) covered with a ribbed black rubbery surface (TSE, Bad Homburg, Germany). Mice received three training sessions (within 45 min) to remain on the rotating rod while it was accelerated from 4 to 40 rpm within 600 s (Paylor et al., 2006). The latency to fall off the rotating drum was recorded.

### Hormone analysis

For corticosterone and adrenocorticotrophic hormone (ACTH) measurements, naïve NgR2^+/+^, NgR2^+/−^, and NgR2^−/−^ animals were decapitated between 8:00 and 12:00 am. Trunk blood samples were immediately collected and centrifuged at high speed to separate the plasma and stored at −20°C until the assay was carried out. Plasma corticosterone and ACTH concentrations were measured with radioimmunoassay kits (MP Biomedicals) according to the manufacturer's instructions. The manufacturer-reported intra-assay and inter-assay coefficient of variation were ≤10% for all kits used. Two separate cohorts of animals were used and corticosterone and ACTH values were normalized to NgR2^+/+^ in both cohorts before being pooled for analysis. This resulted in a total *n* of 8–9 animals/genotype for ACTH measurements and 9 animals/genotype for corticosterone measurements.

### Statistics

Statistical analysis was conducted using SPSS or Statistica. Morphological data and hormone data were analyzed using student's *t*-test to compare genotypes. Behavioral studies were analyzed using student's *t*-test, One-Way ANOVA, or Two-Way repeated measures ANOVA, unless noted otherwise. *Post-hoc* comparisons were performed using Fisher's protected least significant difference (Fisher's PLSD) multiple comparisons. Statistical significance was set at *p* < 0.05. Values in graphs are expressed as mean ± s.e.m.

## Results

### Loss of NgR2 alters distribution of spine morphologies in the hippocampus

**Figure 1:**
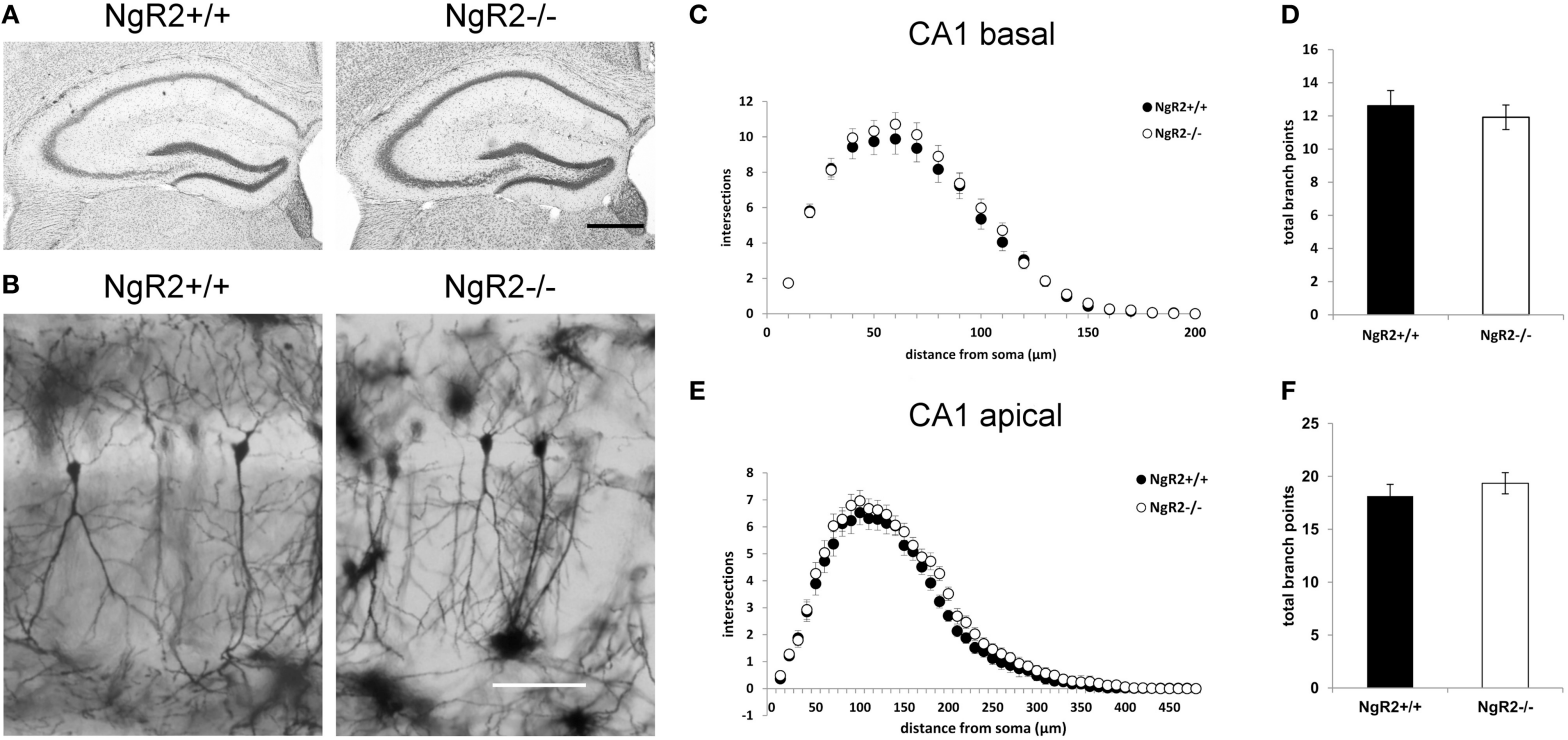
**Dendritic morphology of CA1 hippocampal neurons is similar between NgR2^+/+^ and NgR2^−/−^ mice. (A)** Nissl staining of coronal sections from NgR2^+/+^ and NgR2^−/−^ hippocampus. **(B)** Representative images of Golgi-Cox stained pyramidal cells in hippocampal CA1 region in NgR2^+/+^ and NgR2^−/−^. **(C,D)** Sholl analysis of dendritic complexity in CA1 basal dendrites. Number of intersections were similar between genotypes **(C)**, and the total number branch points were unaltered between genotypes **(D)**. **(E,F)** Sholl analysis of dendritic complexity in CA1 apical dendrites. Number of intersections **(E)** and total branch points **(F)** were unaltered between genotypes. Scale bars: **(A)** 200 μm, **(B)** 100 μm.

In order to study the effects of NgR2 on adult neuronal plasticity, we used adult (8–10 week) NgR2^−/−^ and NgR2^+/+^ littermate controlled mice, generation of which has been previously detailed (Wörter et al., 2009). Anatomy of Nissl-stained NgR2^−/−^ brains appeared normal compared to littermate controls (Figure 1A). Neuronal density in the hippocampus was analyzed using immunohistochemistry for the neuron-specific marker NeuN. No significant change in the density of NeuN-positive cells was observed in the hippocampal CA1 region (mean NeuN positive cells counted in 40,000 μm^2^ area of CA1: NgR2^+/+^ = 72.8 ± 1.7 cells, NgR2^−/−^ = 71.6 ± 1.0 cells; *t* = 0.62, *p* = 0.56). Constitutive knockout of all three NgRs increases hippocampal dendritic complexity *in vivo* at P18 during development (Wills et al., 2012), but it is not known if loss of NgR2 function alone can influence dendritic geometry and spine density in adult neurons. We used Golgi-Cox staining to visualize individual CA1 pyramidal neurons in adult mice (Figure 1B), and analyzed their dendritic trees. Sholl analysis of reconstructed CA1 neurons from NgR2^+/+^ and NgR2^−/−^ mice showed no genotype differences in complexity of basal and apical dendritic trees, as measured by the number of intersections over distance (Figures 1C,E) and the number of branching points per dendrite (Figures 1D,F, Table 1). There was also no effect of genotype on the total dendritic length or the number of dendritic endings in basal and apical dendrites (Table 1). Thus, these observations indicate little, if any, change in overall dendritic structure in NgR2^−/−^ mice.

**Table 1:** **Morphological analysis of apical and basal dendrites in CA1 hippocampal cells from NgR2^+/+^ and NgR2^−/−^ animals**.

**Genotype**	**Apical**			**Basal**		
	**NgR2^+/+^**	**NgR2^−/−^**	***p*-value**	**NgR2^+/+^**	**NgR2^−/−^**	***p*-value**
Total no of intersections	102.1 ± 5.5	114.1 ± 5.5	0.1	85.7 ± 5.9	80.6 ± 5.1	0.5
Total dendritic length (μm)	1541 ± 89	1675 ± 84	0.3	1124 ± 79	1132 ± 67	0.9
Total no of branch points	18.1 ± 1.1	19.3 ± 1.0	0.4	12.6 ± 0.9	11.9 ± 0.7	0.6
Total no of branch tips	19.4 ± 1.1	20.9 ± 1.0	0.4	16.7 ± 1.0	16.3 ± 0.8	0.7

Per genotype and parameter, mean ± s.e.m was determined by averaging values per cell, and these were compared with an independent samples t-test (far right column). N = 7 animals/genotype, n = 74–83 cells/genotype apical, n = 42–49 cells/genotype basal.

**Figure 2:**
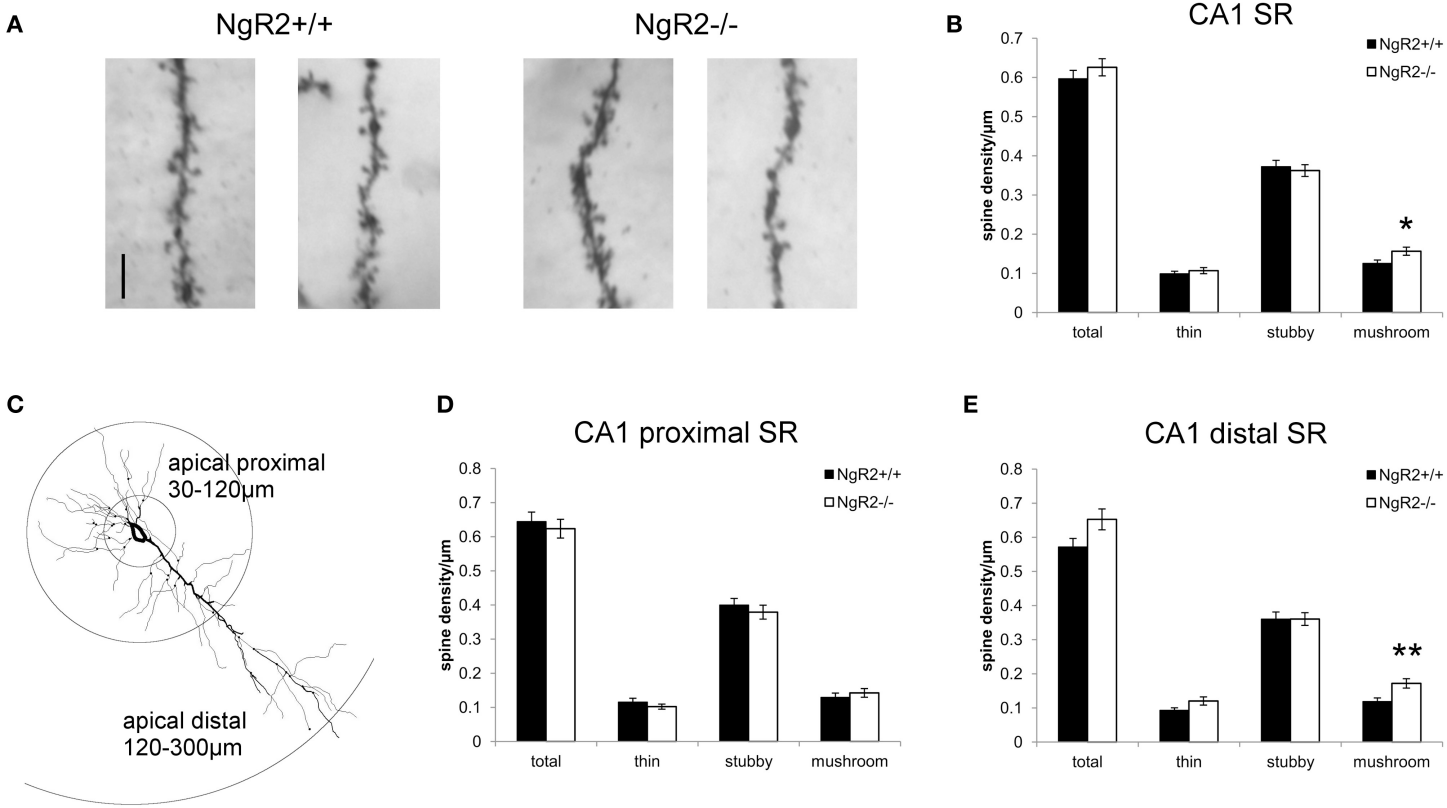
**NgR2^−/−^ mice have an altered distribution of dendritic spine morphologies in CA1 hippocampal neurons. (A)** Representative images of dendritic spines of adult NgR2^+/+^ and NgR2^−/−^ CA1 apical tertiary dendrites from Golgi-Cox staining. **(B)** Quantification of spine densities for each morphological criteria in CA1 apical tertiary dendrites revealed a significant increase in mushroom type spines in NgR2^−/−^ compared to NgR2^+/+^. **(C)** Example of a CA1 pyramidal neuron reconstructed in Neurolucida explorer (3D tracing flattened to 2D), with definition of apical proximal and apical dendritic segments used to divide CA1 stratum radiatum (SR) for spine analysis in **(D,E)**. **(D)** Quantification of spine densities in apical proximal dendritic branches of CA1 SR. **(E)** Quantification of spine densities in apical distal dendritic branches of CA1 SR. A significant increase in mushroom spines was seen in NgR2^−/−^ compared to NgR2^+/+^. *n* = 7 mice, ^*^p < 0.05, ^**^p < 0.005. Scale bar: **(A)** 5 μm.

However, a more detailed analysis revealed striking differences in the distribution of spine sub-types on apical dendrites of CA1 pyramidal neurons. Spines from 2 to 6 tertiary dendritic segments per neuron were analyzed to obtain the averaged spine density for 54–60 CA1 pyramidal neurons per genotype (Figure 2A). Quantitative measures show a significant increase in the density of mushroom-type dendritic spines in NgR2^−/−^ compared to NgR2^+/+^ (mushroom spines NgR2^+/+^ = 0.13 ± 0.01 spines/μm, NgR2^−/−^ = 0.16 ± 0.01 spines/μm; *t* = −2.288, *p* = 0.02), whereas thin and stubby type spines displayed similar densities between genotypes (Figure 2B). This increase did not, however lead to changes in total spine density between NgR2^+/+^ and NgR2^−/−^ (NgR2^+/+^ = 0.60 ± 0.02 spines/μm, NgR2^−/−^ = 0.63 ± 0.02 spines/μm; *t* = −0.951, *p* = 0.34; Figures 2A,B).

In the intact hippocampus, CA1 pyramidal neurons receive spatially segregated direct and indirect excitatory inputs from different anatomical sources (Spruston and McBain, 2007; Kajiwara et al., 2008). Schaffer collateral input from CA3 terminates on CA1 dendrites in both SR (apical dendrites) and stratum oriens (basal dendrites). The majority of input to CA1 apical dendrites is to the distal two-thirds of SR (Bannister and Larkman, 1995), and fewer synapses are made onto dendritic segments in the proximal SR region (Megías et al., 2001). Therefore, to measure spine density in the region of CA1 apical dendrites receiving the majority of Schaffer collateral input, we reanalyzed the spine data to take account of distance of dendritic segment from the soma. Spine morphology and spine density analysis were confined to the tertiary dendritic segments of the CA1 proximal SR (30–120 μm from the cell soma), and distal SR at distances 120–300 μm from the cell soma (Figure 2C), similar to previously published classifications (Perez-Cruz et al., 2011; Pillai et al., 2012). No genotype alterations in total spine density nor in the proportion of morphological sub-types were seen within the proximal CA1 SR subfield (total *t* = 0.27, *p* = 0.60; thin *t* = 1.0, *p* = 0.32; stubby *t* = 0.13, *p* = 0.48; mushroom *t* = 0.30, *p* = 0.48; Figure 2D). In contrast, a significant increase in mushroom type spines in NgR2^−/−^ was quantified in the distal SR, where proportion of mushroom spines increased by 41.6% compared to NgR2^+/+^ (NgR2^+/+^ = 0.12 ± 0.01 spines/μm, NgR2^−/−^ 0.17 ± 0.01 spines/μm; *t* = 0.61, *p* = 0.004, Figure 2E). Interestingly, this increase in mushroom spines in distal SR was not accompanied by an alteration in stubby spines (stubby *t* = 0.94 *p* = 1.0), and whilst a trend toward increased thin spines was observed, it was not statistically significant (thin *t* = 0.001 *p* = 0.056). This was reflected in the slight but non-significant increase in total dendritic spine density in NgR2 knockout mice (NgR2^+/+^ 0.57 ± 0.02 spines/μm, NgR2^−/−^ 0.65 ± 0.03 spines/μm; *t* = 0.088 *p* = 0.053; Figure 2E). Moreover, the increase was not attributable to changes in dendritic diameter of tertiary segments analyzed (NgR2^+/+^ = 0.47 ± 0.03 μm, NgR2^−/−^ = 0.43 ± 0.01 μm, *t* = 1.09 *p* = 0.29). We thus conclude that NgR2 has a role in regulating maintenance of mature spines in adult hippocampal neurons.

### NgR2^−/−^ mice have reduced fear expression and anxiety-like behavior

Changes in spine density in CA1 have been reported to be correlated with stress exposure (Shors et al., 2001; Pawlak et al., 2005; Donohue et al., 2006), learning and memory (Moser et al., 1994, 1997), and anti-depressant effects (Wang et al., 2013) in rodents. Moreover, given that activity-regulated genes are implied to contribute to plasticity (Leslie and Nedivi, 2011), and the recent suggestions that NgRs are regulated by various forms of activity stimuli (Guo et al., 2013; Karlsson et al., 2013), we next examined whether the alterations in spine distribution seen in NgR2^−/−^ mice may also affect behavioral tasks in the adult, such as learning and memory.

**Figure 3:**
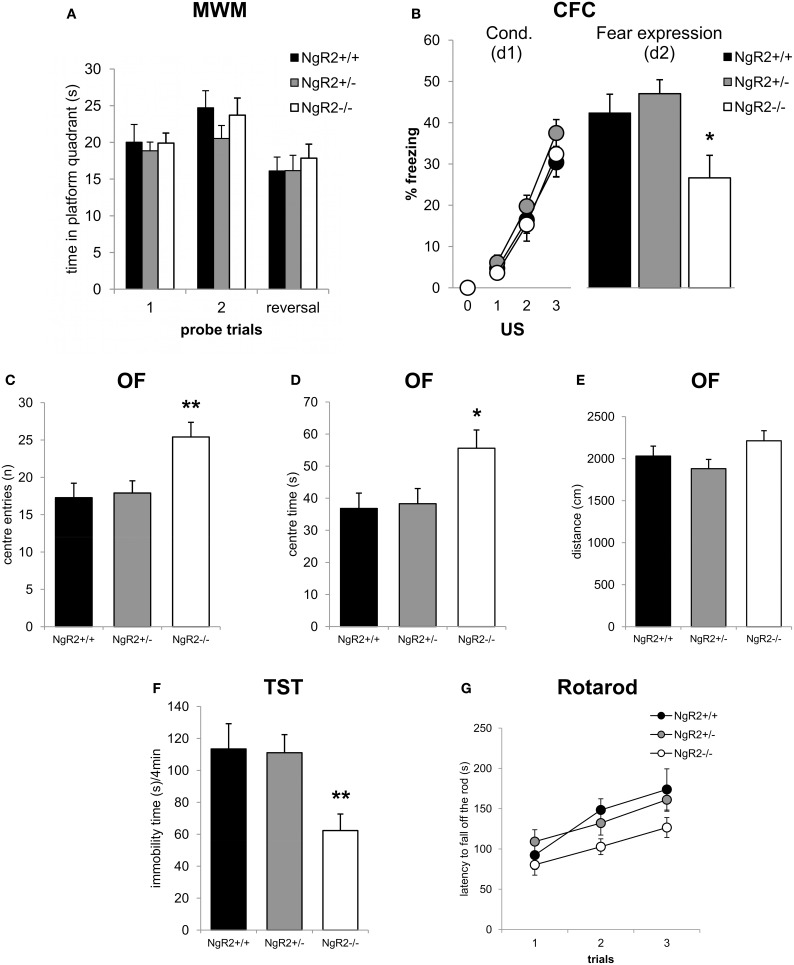
**Altered emotionality in NgR2^−/−^ mice. (A)** In the Morris water maze NgR2^+/+^, NgR2^+/−^, and NgR2^−/−^ did not differ in the time spent in the platform quadrant of the maze during 2 probe trials and a reversal trial. **(B)** Contextual fear conditioning. NgR2^+/+^, NgR2^+/−^, and NgR2^−/−^ mice show similar acquisition of the task, measured by increased freezing behavior over 3 presentations of the unconditioned stimulus (US) in the context. On d2, exposure to the context alone elicited reduced freezing behavior in NgR2^−/−^ compared to NgR2^+/+^ and NgR2^+/+^. **(C–E)** In the open field, NgR2^−/−^ mice had more center entries **(C)** and spent increased time in the center **(D)** compared to NgR2^+/+^ and NgR2^+/−^, without any changes in distance traveled in the arena **(E)**. **(F)** NgR2^−/−^ mice exhibited decreased immobility time in the last 4 min of the tail suspension test. **(G)** In the rotarod test no genotype differences were seen in latency to fall off the rod over 3 trials. *n* = 8-16 for NgR2^+/+^ mice, *n* = 8-14 for NgR2^+/−^, and *n* = 8-15 for NgR2^−/−^ mice. ^*^p < 0.05 and ^**^p < 0.01 NgR2^−/−^ vs. NgR2^+/+^ (analyzed by ANOVA and post Fisher's LSD test).

To test spatial learning and memory, mice were tested in the hidden platform test of the Morris water maze. NgR2^−/−^ mice showed no alterations in learning of the spatial navigation task, as measured by the time spent in the correct quadrant of the pool [genotype effect: *F*_(2, 32)_ = 0.19, *p* = 0.83; genotype × trial: *F*_(2, 32)_ = 0.02, *p* = 0.98; Figure 3A]. In a reversal learning trial there were also no genotype differences in ability to learn a new hidden platform location [*F*_(2, 33)_ = 0.57, *p* = 0.57; Figure 3A]. No deficits in swimming ability were seen (data not shown). To further explore differences in memory between NgR2^+/+^ and NgR2^−/−^ animals, a separate cohort of mice were tested for contextual fear conditioning, a form of classical conditioning that is hippocampal dependent (Phillips and Ledoux, 1992; Anagnostaras et al., 2001). Fear acquisition as indicated by increases in freezing behavior in response to repeated context-shock exposures was normal in NgR2^−/−^ compared to NgR2^+/+^ and NgR2^+/−^ [genotype effect: *F*_(2, 35)_ = 1.27; *p* = 0.29; Figure 3B]. Since the three groups of mice displayed comparable increases in freezing [conditioning effect: *F*_(3, 105)_ = 104.94, *p* < 0.001; conditioning × genotype interaction: *F*_(6, 1105)_ = 0.53, *p* = 0.78], it is suggested that there was no sensorimotor performance deficit, such as an inability to freeze. This is also supported by results from the flinch-jump test in another separate cohort of mice, which showed no differences between NgR2^−/−^ and NgR2^+/+^ in their threshold to flinch (NgR2^+/+^ = 0.10 ± 0.0 mA, NgR2^−/−^ = 0.13 ± 0.03 mA, *t* = 0.36, *p* = 0.36), to jump (NgR2^+/+^ = 0.23 ± 0.06 mA, NgR2^−/−^ = 0.18 ± 0.08 mA, *t* = 0.51, *p* = 0.62) or to vocalize (NgR2^+/+^ = 0.13 ± 0.03 mA, NgR2^−/−^ = 0.25 ± 0.08 mA, *t* = 1.81, *p* = 0.12). Fear expression 24 h later differed between genotypes [*F*_(2, 35)_ = 4.48, *p* = 0.02] as it was significantly reduced in NgR2^−/−^ compared to NgR2^+/+^ (*p* = 0.03) and NgR2^+/−^ (*p* = 0.006; Figure 3B). Reduced freezing could indicate either impairments in memory or impairments in fear response. As we do not detect differences in other tests of spatial and associative memory, this suggests that the reduced freezing seen in contextual fear conditioning may well be due to an altered fear response.

As alterations in anxiety have been shown to be coincident with changes in fear expression (Sartori et al., 2011), we also assessed anxiety-like behaviors in NgR2^−/−^ mice. In the open field test, NgR2^−/−^ showed a significant increase in exploratory behavior compared to NgR2^+/+^ and NgR2^+/−^, as measured by the number of entries [*F*_(2, 33)_ = 6.03, *p* = 0.01, Figure 3C] and time [*F*_(2, 33)_ = 4.18, *p* = 0.02, Figure 3D] spent in the aversive center area, which is usually thought to reflect reduced anxiety (Miyakawa et al., 2001). Locomotor activity in the open field was similar across genotypes, as measured by the total distance traveled [*F*_(2, 33)_ = 1.97, *p* = 0.16; Figure 3E]. In the tail suspension test, NgR2^−/−^ exhibited less immobility compared to littermate controls [*F*_(2, 23)_ = 6.15, *p* = 0.007, Figure 3F], indicative of a decrease in depression-like behavior. This finding fits in the context of the high co-morbidity between clinical disorders of anxiety and depression, which is also reflected in the overlap between tests used to model symptoms of these disorders in rodents (Cryan and Holmes, 2005). In addition to measuring activity in the open field, motor abilities of mice were also tested on the rotarod. No significant alterations in the performance in the rotarod task between genotypes were observed [genotype effect: *F*_(2, 31)_ = 2.95, *p* = 0.07; trial × genotype interaction: *F*_(4, 62)_ = 1.22, *p* = 0.31; Figure 3G], thus it seems unlikely that alterations in the open field exploration and tail suspension test in NgR2^−/−^ mice were due to abnormalities in locomotor activity.

**Figure 4:**
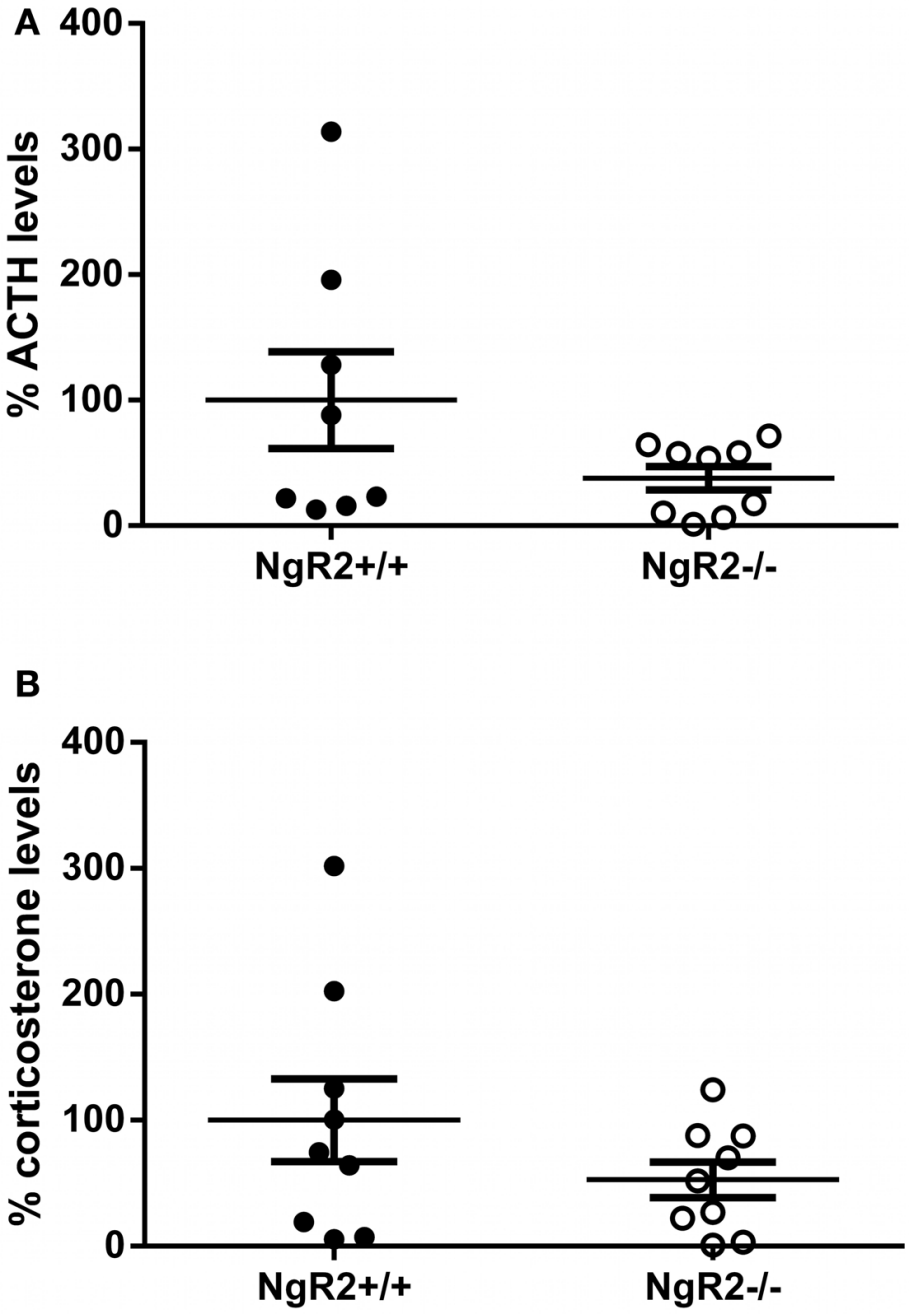
**Basal ACTH and corticosterone levels in NgR2^−/−^ mice. (A)** Basal ACTH measurements normalized to NgR2^+/+^. **(B)** Basal corticosterone measurements normalized to NgR2^+/+^. *n* = 8–9/genotype for ACTH, *n* = 9/genotype for corticosterone, data pooled from 2 cohorts of animals.

The phenotype of increased anxiolytic and reduced fear behavior led us to wonder if NgR2^−/−^ mice were more resilient to stress, leading to better performance under the stressful circumstances of behavioral testing. Basal levels of stress hormones have been reported to correlate with performance in tests of anxiety-like and depression-like behavior in mouse models of stress reactivity (Wittmann et al., 2009; Chourbaji et al., 2010). Therefore, the basal concentrations of ACTH and corticosterone were measured in naïve NgR2^+/+^ and NgR2^−/−^ mice. NgR2^−/−^ mice seemed to have reduced basal ACTH (NgR2^+/+^ = 100.0 ± 38%, NgR2^−/−^ = 37.8 ± 9.4%) and corticosterone (NgR2^+/+^ = 100.0 ± 33%, NgR2^−/−^ = 52.7 ± 14.2%) concentrations compared to NgR2^+/+^, but due to high variability, this did not reach statistical significance (ACTH: *t* = 1.66, *p* = 0.12; CORT: *t* = 1.32, *p* = 0.21; Figure 4). These results indicate that NgR2^−/−^ mice have reduced fear and anxiety-type behaviors without a significant shift of basal hypothalamic-pituitary-axis activity.

## Discussion

In this study we show for the first time that constitutive knockout of NgR2 can alter spine morphology in the adult hippocampus, increasing mushroom-type spines on apical dendrites of CA1 neurons, specifically in distal SR. Coincident with this, absence of NgR2 results in reduced fear, anxiety- and depression-related behaviors. We conclude that NgR2 is involved in maintenance of mature spines in addition to its role in restricting synapse formation.

In the adult system, a role for NgR1 in regulating synaptic turnover has been elucidated, but comparatively little is known about NgR2. NgR1 knockout resulted in a shift of spine morphologies in CA1 apical dendrites, with density of stubby spines increased and mushroom and thin spines decreased (Lee et al., 2008; Delekate et al., 2011), and recent work has shown that in the cortex, spine turnover in NgR1 knockout mice is significantly increased (Akbik et al., 2013). This implies regulation of spine motility by NgR1. Our findings suggest that NgR2 also has a role in regulation of the morphology of dendritic spines. We find that knockout of NgR2 increases mushroom type spines on distal apical dendrites in the adult CA1, whilst not significantly altering proportions of thin or stubby spines. Here it should be noted that relative proportions of spine subtypes reported in fixed tissue in the CA1 region vary in the literature. We report here a higher proportion of stubby spines relative to mushroom spines, which has also been observed by Beltrán-Campos and co-workers and Sanders and co-workers (Beltrán-Campos et al., 2011; Sanders et al., 2012), whereas other studies have found the reverse ratio (Rauskolb et al., 2010; Görlich et al., 2012), or comparatively equal proportions of both subtypes (Lee et al., 2008). These differences are most likely accounted for by methodological differences between studies, particularly with regards to whether spine subtype characterization was carried out with computational or observational methods, and the dendritic branch order of analyzed spines.

Mushroom-type spines have been shown to have an increased number of AMPA receptor subunits (Matsuzaki et al., 2001; Matsuo et al., 2008) and a larger post-synaptic density, which increases their spine head size and are hallmarks of excitatory, or asymmetric synapses (Sheng and Hoogenraad, 2007). Our results, combined with the findings that *in vitro* NgR2^−/−^ hippocampal neurons have increased excitatory synapses (Wills et al., 2012), raises the possibility that this could correspond to an increase in excitatory synapses in adult NgR2^−/−^ mice, which requires further investigation. Whilst we show here that adult spine morphology is altered, it is possible that constitutive loss of NgR2 affects spine morphology already during development. In juvenile mice lacking both NgR1 and NgR2, no differences in CA1 spine density are seen (Wills et al., 2012), but changes in spine subtype distribution at this developmental stage have not been studied.

Interestingly, however, conditional deletion of NgR1 in aged mice increased spine turnover of cerebral cortex neurons to the same level as seen in constitutive NgR1^−/−^ mice in adulthood (Akbik et al., 2013), suggesting a role of NgR1 in stabilizing dendritic spines in the adult cortex. However, it remains to be seen if the effect of NgR2 on spine maturation is temporally specific to developmental or adult windows of plasticity.

Spine morphogenesis involves both spine motility and spine stabilization. The protrusive motility of filopodia and stabilization of spines by head morphing have been demonstrated to be separately regulated stages of spinogenesis (Tashiro and Yuste, 2004). Applying this model to our data on NgR2 would suggest that NgR2 might be involved in processes to inhibit the morphing of immature spine subtypes into mushroom-type spines, and therefore regulation of spine stability. This would suggest that NgR2 is not required for spine motility, as NgR1 is, although further experiments must be done to investigate this.

NgR1 has been shown to be located on dendrites and to be enriched pre- and post-synaptically (Wang et al., 2002b; Lee et al., 2008; Raiker et al., 2010; Wills et al., 2012). However, it is not confirmed if NgR2 also is present at synaptic sites. Its presence in the human post-synaptic density has been suggested by a recent study, although it was not consensus in all samples (Bayés et al., 2012). Due to the absence of a good antibody against this receptor, more precise sub-cellular localization is currently lacking, though there is suggestion of dendritic localization in hippocampal cultures, in a similar pattern to that seen with NgR1 (Wills et al., 2012). The region-specific differences we observed in spine morphology, with increased mushroom spines in distal SR but no differences in proximal SR, could be due to differential localization of NgR2 across pyramidal neurons. Another possibility is that alterations are only seen in distal SR due to input-specific regulation of synaptic function. The increase in mushroom type spines in distal SR, suggest that knockout of NgR2 might be affecting synaptic efficacy at excitatory Schaffer collateral input from CA3. Given the role already described for NgR1 in negatively regulating LTP at Schaffer collaterals (Lee et al., 2008; Delekate et al., 2011), this warrants further investigation. Limitations of the Golgi-Cox method mean we were not able to analyze dendritic spine density throughout the full extent of SLM, so we cannot exclude that spine differences are also observed in this region, which receives direct input from entorhinal cortex and basolateral amygdala, as well as thalamic and septal projections. Possible functional redundancy of NgRs should also be considered. The effects observed in spine density in NgR2 null mutants, as well as NgR1 null mutants (Lee et al., 2008), are small compared to that seen in NgR triple knockout mice (Wills et al., 2012). Another recent study using NgR triple knockout mice also suggests a functional redundancy effect of these receptors in regeneration after injury (Dickendesher et al., 2012).

Observations of reduced fear and anxiety alongside CA1 specific spine alterations in NgR2^−/−^ mice is of particular note due to the involvement of the CA1 region of the hippocampus in mediating anxiety and fear behaviors. Inactivation of dorsal CA1 with the GABA_A_ agonist muscimol increased anxiolytic response in the elevated plus maze (Rezayat et al., 2005). Lesions to dorsal CA1 or transaction of CA1 efferents to the septum indicated that dorsal CA1 was involved in both the encoding and retrieval of contextual fear (Hunsaker and Kesner, 2008; Hunsaker et al., 2009). Additionally, fear and anxiety behaviors have been shown to alter dendritic spine density in the CA1 region. An anxiolytic phenotype was seen after repeated injection with the TCAP-1, a teneurin C-terminal associated peptide, and in the same animals increases in spine density were seen in apical dendrites in CA1 SR and SLM (Tan et al., 2011). In mice selectively bred for high or low stress reactivity, spine density was increased in distal SR of low stress reactive mice, without alterations in dendritic complexity (Pillai et al., 2012). Fear learning can recruit new mushroom-type spines in the hippocampus (Matsuo et al., 2008), and spine density is increased in the hippocampal CA1 region 24 h after contextual conditioning (Pignataro et al., 2013). Depression-like behavior is also associated with spine alterations. Correlations between learned helplessness and spine synapses suggests that behavioral despair decreases as spine density increases (Hajszan et al., 2009). Additionally, anxiolytic and anti-depressant behavioral effects has been shown to be associated with enhanced neuronal activity in the hippocampus (Dagyte et al., 2010; Sah et al., 2012). The behavior phenotype observed here with NgR2^−/−^ mice is distinct from that seen when NgR1 is modulated. NgR1 knockout studies have shown impaired working memory (Budel et al., 2008) and improved extinction learning (Akbik et al., 2013), but no effects on anxiety have been reported. This suggests alterations in anxiety are unique to a loss of NgR2.

There is evidence that NgR1 may be an activity-regulated gene, as its downregulation at mRNA level has been demonstrated after various neuronal activity inducing stimuli, such as running and kainic acid (Josephson et al., 2003; Mingorance et al., 2004; Karlsson et al., 2013). It is still not clear if NgR2 is also an activity regulated gene. *In vitro* studies in hippocampal neuronal cultures indicate neuronal depolarization, by KCl or NMDA treatment, decrease mRNA expression of NgR1–3 (Wills et al., 2012). However, an *in vivo* study following the time course of NgR2 expression after kainic acid challenge showed that the fast and transient downregulation of NgR1 by 2 h is then followed by upregulation of NgR2 and NgR3 in the dentate gyrus, peaking around 12 h post injection (Karlsson et al., 2013). These temporal differences have led to the suggestion that the immediate downregulation of NgR1 which enables increased plasticity is followed by increases in NgR2 and NgR3 which close the window of plasticity again (Karlsson et al., 2013). Our data suggest a more complex scenario, in which both NgR1 and NgR2 can restrict plasticity, but may have differential effects on synaptic remodeling. Further work is required to understand the temporal dynamic of this regulation. Intriguingly, an *in vivo* study with the stimulant amphetamine observed that both NgR1 and NgR2 protein levels were decreased in the hippocampus (Guo et al., 2013). Clearly there are certain differences between these paradigms that could be underlying differential effects seen with NgR2 expression after neuronal activity is induced. There are differences between *in vitro* and *in vivo* neuronal depolarization paradigms, and amphetamine stimulation affects the dopaminergic system, another point of difference. In the context of differential effects of NgR1 and NgR2 after neuronal activity and in modulation of spine dynamics, it is worth noting differences in ligand binding for these receptors. Of the myelin-associated inhibitors, NgR1 can interact with Nogo-A, oligodendrocyte myelin glycoprotein (OMgp) and myelin-associated glycoprotein (MAG) (Fournier et al., 2001; Domeniconi et al., 2002; Wang et al., 2002a), whilst NgR2 only binds to MAG (Venkatesh et al., 2005). NgR1 additionally has been shown to bind to the glycosaminoglycan (GAG) side chains of chrondroitin sulfate proteoglygans (CSPGs), which NgR2 does not (Dickendesher et al., 2012), and additionally, NgR1 and NgR2 both interact with amyloid precursor protein (APP) via interference with β-secretase 1 (BACE-1) cleavage (Park et al., 2006; Zhou et al., 2011). It is likely that other as yet unidentified ligands interact with NgR2 besides MAG, and such unknown ligands may be modulating aspects of NgR2 signaling in the CA1 region which influence spine dynamics. Further investigation of how regulation of NgRs after neuronal activity is related to binding of specific ligands may also shed light on mechanisms required for NgR2 signaling in regulating spine morphology.

We conclude that NgR2 can function to regulate mature spine morphology, and alter fear and anxiety-related, as well as depression-related, behavior. Further work will be needed to elucidate if these alterations in spines are contributing directly to modifying behavior. Furthermore, it will be important to reveal which ligands of NgR2 are involved in mediating this effect, to determine if modulating Nogo receptor 2 signaling can provide further insights into regulating anxiety-like behaviors.

## Author contributions

Sarah C. Borrie, Simone B. Sartori, Nicolas Singewald, and Christine E. Bandtlow designed the experiments. Sarah C. Borrie, Simone B. Sartori, and Julian Lehmann performed the experiments. Sarah C. Borrie, Simone B. Sartori, and Anupam Sah analyzed the data. Sarah C. Borrie and Christine E. Bandtlow wrote the paper.

### Conflict of interest statement

The authors declare that the research was conducted in the absence of any commercial or financial relationships that could be construed as a potential conflict of interest.
